# Use of Botulism Antitoxin Heptavalent (A, B, C, D, E, F, G)—(Equine) (BAT^®^) in Clinical Study Subjects and Patients: A 15-Year Systematic Safety Review

**DOI:** 10.3390/toxins14010019

**Published:** 2021-12-27

**Authors:** Geraldine S. Parrera, Hugo Astacio, Priya Tunga, Deborah M. Anderson, Christine L. Hall, Jason S. Richardson

**Affiliations:** 1 Pharmacovigilance Department, Emergent BioSolutions Canada Incorporated (Inc.), Winnipeg, MB R3T 5Y3, Canada; gparrera@ebsi.com (G.S.P.); hastacio@ebsi.com (H.A.); 2 Biostatistics Department, Emergent BioSolutions Canada Inc., Winnipeg, MB R3T 5Y3, Canada; priyadarshini.raghavendra@gmail.com (P.T.); andersond@ebsi.com (D.M.A.); 3 Clinical Research Department, Emergent BioSolutions Canada Inc., Winnipeg, MB R3T 5Y3, Canada; chall@ebsi.com

**Keywords:** safety review, equine-derived, Botulism Antitoxin Heptavalent, BAT product, botulism

## Abstract

Botulism is a rare, sometimes fatal paralytic illness caused by botulinum neurotoxins. BAT^®^ (Botulism Antitoxin Heptavalent (A, B, C, D, E, F, G)—(Equine)) is an equine-derived heptavalent botulinum antitoxin indicated for the treatment of symptomatic botulism in adult and pediatric patients. This review assesses the cumulative safety profile for BAT product from 2006 to 2020, using data received from clinical studies, an expanded-access program, a post-licensure registry, spontaneous and literature reports. The adverse event (AE) incidence rate for BAT product was calculated conservatively using only BAT product exposures for individuals with a record (512) and was alternatively estimated using all BAT product exposure data, including post-licensure deployment information (1128). The most frequently reported BAT product-related AEs occurring in greater than 1% of the 512–1128 BAT product-exposed individuals were hypersensitivity, pyrexia, tachycardia, bradycardia, anaphylaxis, and blood pressure increase reported in 2.3–5.1%, 1.8–3.9%, 1.0–2.2%, 0.89–2.0%, 0.62–1.4%, and 0.62–1.4%, respectively. For patients properly managed in an intensive care setting, the advantages of BAT product appear to outweigh potential risks in patients due to morbidity and mortality of botulism. AEs of special interest, including bradycardia, hemodynamic instability, hypersensitivity, serum sickness, and febrile reactions in the registry, were specifically solicited.

## 1. Introduction

Botulism is caused by botulinum neurotoxin (BoNT) produced by several members of the genus *Clostridium* and mostly by *Clostridium botulinum*, a spore-forming bacillus bacteria widely found in soils [1,2]. There are several antigenically distinct BoNT serotypes, designated by the letters A through G, and while humans are susceptible to all of them, botulism is usually associated with serotypes A, B, E, and F [3,4]. Recently, BoNT X and BoNT/H were identified as a unique branch of the botulinum neurotoxin family [5,6]. The neurotoxins irreversibly block the release of acetylcholine at the neuromuscular junction, leading to illness characterized by descending paralysis [7]. If untreated, involvement of the respiratory musculature can lead to respiratory failure and death [8].

Botulism presents clinically in six recognized forms, including foodborne, infant, intestinal, wound, iatrogenic, and inhalation botulism [9]. Each form is characterized by different exposure routes and incubation periods between contamination and the onset of symptoms [9]. Naturally occurring cases of botulism are rare in humans. Based on available data from the United States of America (U.S.) Centers for Disease Control and Prevention (CDC), from 2013 to 2017, there were 900 confirmed cases and 15 deaths reported due to botulism with an average of 180 cases per year (minimum 153 cases in 2013, maximum 205 cases in 2016) [10]. In Europe, 547 confirmed cases and 17 deaths were reported in the same time frame, with an average of 109 cases per year (minimum 86 cases in 2017, maximum 128 cases in 2016) [11]. Botulinum neurotoxin could potentially be used in an intentional bioterrorism attack due to its extreme toxicity as a point source aerosol [12,13]. In the U.S., botulism is a notifiable disease, allowing health authorities to detect, monitor, and address possible outbreaks and intentional exposures [14].

There are two U.S.-licensed products for the treatment of botulism: BabyBIG^®^ is a human immunoglobulin product used to treat infant botulism (less than one year of age) caused by BoNT serotypes A and B [15]. Botulism Antitoxin Heptavalent (A, B, C, D, E, F, G)—(Equine), product name BAT, is an equine-derived heptavalent botulinum antitoxin indicated for the treatment of symptomatic botulism following documented or suspected exposure to BoNT serotypes A, B, C, D, E, F, or G in both adult and pediatric patients [16]. BAT product was approved by the U.S. Food and Drug Administration (FDA) in 2013, by Health Canada in 2016, by Singapore and Ukraine in 2019 [17,18,19]. BAT product is made available to other countries through country-specific special access or emergency use programs. Currently, in the U.S., BAT product is part of the U.S. Strategic National Stockpile (SNS) and is available emergently and free of charge from the federal government to treat suspected botulism, following emergency clinical consultations with health departments and the CDC [4,20]. In Canada, BAT product is kept either at a provincial depot or by the Public Health Agency of Canada’s National Emergency Stockpile System (NESS) and is released for use following consultation with the applicable provincial or federal public health official [21]. Therefore, the collection of safety data for BAT product has been robust, due in part to the well-controlled distribution of BAT product.

Emergent BioSolutions Canada Inc. (EBCI) (Winnipeg, Canada) is the manufacturer and license holder of BAT product. The BAT product clinical development program was initiated in healthy volunteers in 2006 and was available for use under a CDC expanded-access program (EAP) under Investigational New Drug Application (IND) from March 2008 through March 2013 [22,23,24]. Under this EAP, one pediatric patient treated with BAT product developed hemodynamic instability without anaphylaxis [24]. This related adverse event (AE) instigated a U.S. FDA post-marketing requirement for EBCI to conduct a three-year BAT product registry with the goal of continued targeted safety surveillance for hemodynamic instability, hypersensitivity reactions, serum sickness, febrile reactions, and bradycardia, termed here as AEs of special interest (AESI) [17,25].

Equine-derived botulism antitoxins have been commercially available in the U.S. since the 1940s [26]. Based on historical data for human use, the principal risk of equine hyperimmune products is immunologic reactions, including anaphylactic and anaphylactoid reactions, and delayed allergic reactions, including serum sickness and possible febrile responses to immune-complex formation [27]. Since BAT product is derived from pooled equine plasma containing immunoglobulin fragments, it is necessary to assess the incidence of immediate or delayed hypersensitivity reactions (Types I or III, respectively) [28,29,30] in individuals exposed to BAT product.

This work includes a 15-year systemic safety review of BAT product with a particular interest in cases associated with AESI using data from two phase 1 clinical studies, the CDC’s EAP, a three-year post-marketing BAT product registry, and spontaneous and literature reports in the U.S. and in the rest of world (ROW) through to a cutoff date of 21 March 2020, which will better inform clinicians of BAT product safety profile. The questions that guided our systematic review included: (i) What are the related AEs that are seen with BAT product? (ii) What is the incidence of hypersensitivity reactions to BAT product? (iii) What is the incidence of related AESI?

## 2. Results

Data sources included two EBCI-sponsored studies in healthy adult volunteers (BT-001 [23] and BT-002 [22]), a CDC-sponsored EAP under IND [24], a post-licensure observational patient registry (BT-010) [25], and post-marketing spontaneous and literature individual case safety reports received from licensure up to a cutoff date of 21 March 2020 (Table 1). These data sources were used to estimate the total number of BAT product-exposed individuals and to determine the number of individuals who experienced BAT product-related AEs.

### 2.1. Literature Review

**Figure 1:**
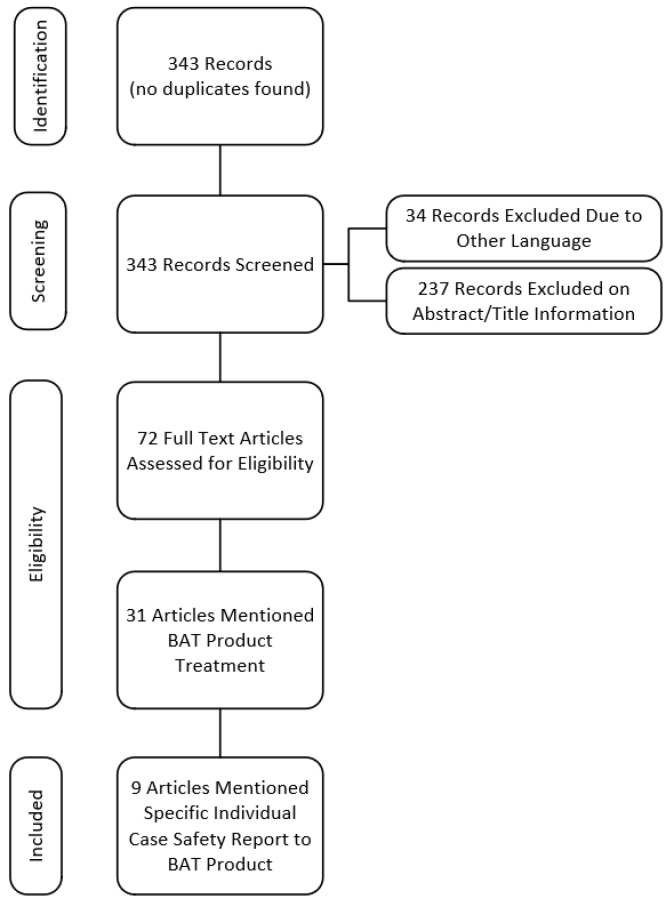
Preferred reporting items for systematic reviews and meta-analyses (PRISMA) flow diagram for literature articles reporting adverse events to BAT product (2005–2020).

The literature search strategy yielded 343 records, 34 non-English publications were excluded, 237 records were excluded based on abstract/title review, and 72 full-text articles were assessed for eligibility (Figure 1). Thirty-one articles mentioned treatment with BAT product. Nine articles mentioning BAT product-related AEs were included in the analysis (see Material & Methods for literature search and article screening strategies).

### 2.2. BAT Product Exposed Population

**Figure 2:**
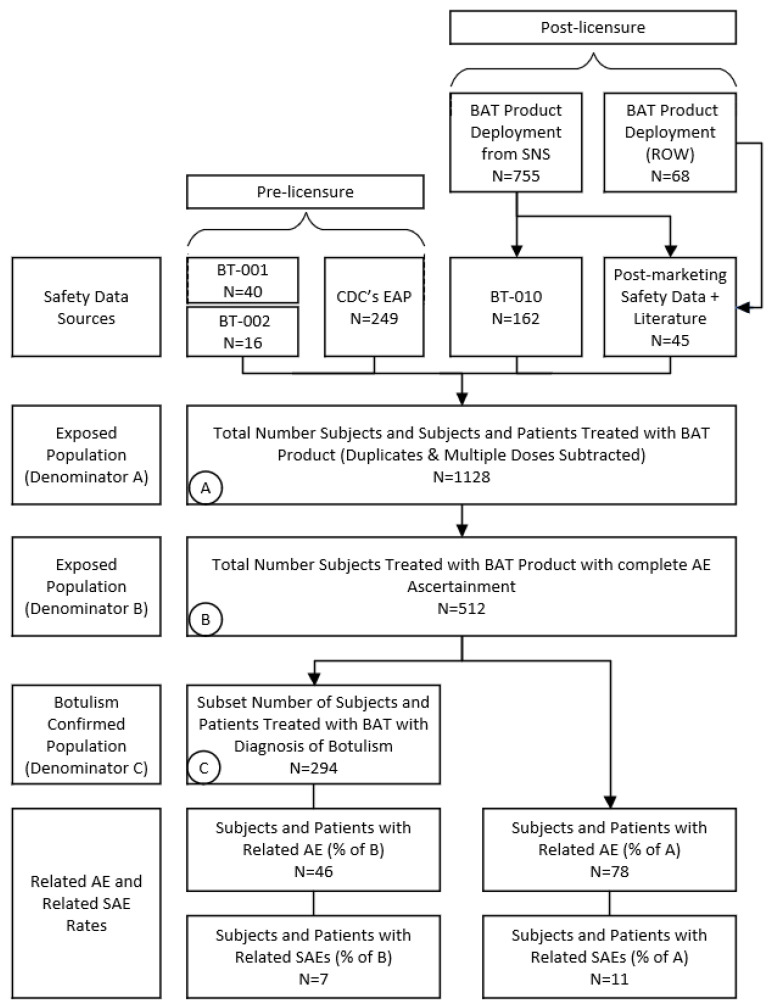
Preferred reporting items for systematic reviews and meta-analyses (PRISMA) flow diagram: total BAT product exposures and incidence of related AEs/SAEs.

Only confirmed BAT product exposures in the pre- and post-U.S. licensure settings (i.e., BT-001, BT-002, CDC’s EAP, BT-010, U.S. CDC BAT product deployment information, ROW BAT product distribution) up to 21 March 2020 were included. Only BAT product-related AEs from the specified data sources are included in this review, as shown in Figure 2 (see Materials & Methods for data eligibility criteria and calculations of AE incidence rate).

### 2.3. Demography

Among the 512 BAT product-exposed individuals aged 32 days to 92 years (median 44 years), 26 (5.1%) were pediatric patients, 377 (73.6%) were adults, and 78 (15.2%) were geriatric (Table 2). A total of 66% were male, and 33.8% were female.

Two hundred ninety-four (294) patients among the 512 BAT product-exposed individuals had a final diagnosis of botulism.

### 2.4. Product-Related Adverse Events Identified in the Master Safety Dataset

The related AEs were categorized by system organ class (SOC) and preferred term (PT) and grouped by population, i.e., individuals with a final diagnosis of botulism vs. healthy volunteers from EBCI phase I studies vs. other (the patients who received BAT product with a non-botulism final diagnosis) (Table 3).

Overall, 78 (15.2%) of the 512 BAT product-exposed subjects or patients reported BAT product-related AEs. A total of 46 (15.7%) of the 294 patients with a final diagnosis of botulism reported BAT product-related AEs. A total of 10 (17.86%) of the 56 BAT product-exposed healthy volunteers from the EBCI phase I studies reported BAT product-related AEs. Of the BAT product-exposed patients with the final diagnosis “not botulism”, 22 (13.6%) patients reported BAT product-related AEs.

Overall, the most frequently reported BAT product-related AEs occurring in greater than 1% of the 512 individuals were pyrexia (20 individuals (3.9%)), tachycardia (11 individuals (2.2%)), bradycardia (10 individuals (2.0%)), and blood pressure increased (7 individuals (1.4%)). Nausea, headache, and urticaria were each reported in 5 (0.98%) individuals (see Table 3 for all other related AEs).

Of the 294 individuals with a final diagnosis of botulism, the BAT product-related AEs that occurred at an incidence of greater than 1% were pyrexia (12 individuals (4.1%)), tachycardia (6 individuals (2.0%)), bradycardia (5 individuals (1.7%)), and blood pressure increased (4 individuals (1.4%)).

Overall, of the 512 individuals administered BAT product, 11 individuals (2.2%) reported BAT product-related serious adverse events (SAEs), including: 3 events of hypersensitivity in 3 individuals (0.59%), 3 events of anaphylactic reaction in 3 individuals (0.59%), 2 events of hemodynamic instability in 2 individuals (0.39%), 1 event in each individual of acute myocardial infarction, ventricular tachycardia, acute kidney injury and 1 individual with anaphylactic shock and distributive shock associated with hemodynamic instability (0.20%) (Table 4).

### 2.5. Product-Related Adverse Events of Special Interest (AESI) Identified in the Master Safety Dataset

Two individuals (0.39%) had hemodynamic instability. A total of 11 hypersensitivity reactions (hypersensitivity, anaphylactic reaction, anaphylactic shock, serum sickness-like reaction, and serum sickness) occurred in 11 individuals (2.1%). Three serious events of anaphylactic reactions occurred in three individuals (0.59%). One serious event of anaphylactic shock was reported in one individual (0.20%). Two non-serious events of serum sickness-like reaction were reported in two individuals (0.39%). One non-serious event of serum sickness was reported in one individual (0.20%). It is difficult to determine whether any of the pyrexia events satisfied the definition of febrile reaction since febrile reactions code to pyrexia. Nonetheless, there were 21 related non-serious AEs of pyrexia in 20 individuals (3.91%). A total of 10 non-serious events of bradycardia were reported in 10 individuals (1.95%).

### 2.6. Integrated Hypersensitivity Assessment

Using the Gell and Coombs’ classification of immunologic drug reactions [31], 26 individuals (5.1%) were identified as having hypersensitivity reactions. Anaphylaxis was reported directly as a diagnosis for four individuals. In our analysis, three additional individuals had reactions that met the Brighton Collaboration Working Group case definition of anaphylaxis [32] (Supplementary Material Text S3); therefore, we determined that seven individuals (1.37%) developed anaphylaxis.

## 3. Discussion

BAT product is an equine-derived product, and the primary safety concerns associated with this drug product class are hypersensitivity reactions (type I (acute) and type III (delayed reactions such as serum sickness)) to these extraneous proteins [27,33,34,35]. Febrile reactions, bradycardia, and hemodynamic instability are adverse events of special interest for BAT product and may or may not be associated with hypersensitivity reactions.

The despeciation procedure performed during the BAT product manufacturing process (pepsin digestion to remove the F_c_ portion of the whole IgG molecule) is designed to reduce these types of reactions [36]. The manufacturing process for each antitoxin serotype includes cation-exchange chromatography to further purify the immune globulin fraction, digestion with pepsin to produce F(ab′)_2_ plus F(ab′)_2_-related immune globulin fragments, anion exchange chromatography to remove the pepsin as well as other impurities and filtration.

Historically hypersensitivity reactions have been reported in 6.5–9.0% of patients receiving equine-derived antitoxin, regardless of age or sex or antitoxin type, where 5.3% were acute reactions and 1–4% were delayed (serum sickness) [27,36,37]. Schussler et al. reported anaphylaxis incidence for BAT product as 1.6%, whereas the incidence for anaphylaxis for other botulinum antitoxins (not-BAT product) as 1.2% [37]. In our analysis, we used two approaches to identify hypersensitivity reactions to BAT product. We used the master data set to find all BAT product related hypersensitivity reactions (hypersensitivity, anaphylactic reaction, anaphylactic shock, serum sickness-like reaction, and serum sickness) and calculated an incidence rate of 2.1% for hypersensitivity reactions, which is similar to the incidence of 1–6.2% in other equine immunoglobulins [38]. However, anaphylactic and hypersensitivity reactions (type 1 and type III) often present as symptoms [34,39] and are not always reported by healthcare professionals or investigators as hypersensitivity to enter into the pharmacovigilance (PV) safety database, Oracle^®^ Argus Safety (Argus). Therefore, hypersensitivity reactions are difficult to characterize, and the use of a standardized Medical Dictionary for Regulatory Activities (MedDRA) query (SMQ) to determine the number of hypersensitivity cases is not always consistent with physician-assessed cases. Thus, we conservatively considered any known hypersensitivity symptoms as a case of hypersensitivity and further evaluated these cases for anaphylaxis using the Brighton Collaboration case definition [32], even though it may have been confounded with other conditions. Using this approach, anaphylaxis, hypersensitivity, and serum sickness were reported in 1.37%, 5.1%, and 0.2% of BAT product-exposed patients, respectively. This anaphylaxis rate aligns with the BAT product anaphylaxis rate reported by Rao et al. [4] and is similar in frequency for previously used formulations [24,27,37,40]. The incidence of serum sickness is similar to that of other despeciated equine antitoxins such as equine rabies immunoglobulin, reported as 0.87% to 6.19% [41,42].

However, because the information received was limited and incomplete, there were some assumptions that were included in the assessment of hypersensitivity. In our analysis, a few hypersensitivity reactions that occurred during BAT product infusion (Supplementary Material Text S3) were resolved by slowing or interrupting the infusion to treat the reaction, thereby preventing progression to anaphylaxis. None of the BAT product-related hypersensitivity reactions resulted in fatal outcomes.

Febrile reactions are common adverse reactions from blood transfusions (including immunoglobulins) [43]. Since febrile reactions code to MedDRA PT “pyrexia”, it was difficult to determine whether the BAT product-related pyrexia events were “febrile reactions” (fever with chills). Nonetheless, there were 21 BAT product-related non-serious AEs of pyrexia in 20 individuals (3.91%).

The causes of hemodynamic instability are cardiogenic, hypovolemic, distributive, and obstructive shock [44]. We observed two (2; 0.39%) BAT product-related cases of hemodynamic instability in our data. One case was secondary to anaphylaxis, and the other case occurred in an individual experiencing asystole during BAT product infusion [24]. In the latter case, the safety database captured the hemodynamic instability as a diagnosis characterized by tachycardia, bradycardia, and asystole.

In our evaluation, 10 (1.95%) individuals had BAT product-related bradycardia. Bradycardia was specifically solicited during active surveillance of the BAT product registry and may have contributed to increased reporting. True bradycardic reactions have multiple causes, including botulism itself [45]. Normal physiological changes in heart rate occur within certain situations, e.g., in endurance training where the heart pumps more blood with less effort. The increase in cardiac parasympathetic activity (vagal tone) is a major contributor [46]. The pathophysiology of sinus bradycardia depends on the underlying cause.

Four unexpected, related SAEs: acute myocardial infarction, ventricular tachycardia, acute kidney injury, and distributive shock were reported. Acute myocardial infarction (MI), reported as a mild non-ST-elevation myocardial infarction (NSTEMI), occurred in a patient with confirmed botulism complicated with pulmonary edema and aspiration pneumonia and concurrent coronary artery disease (CAD) and hypertension, two days after BAT product infusion. Acute MI resolved with triple vessel heart catheterization and was considered BAT product related despite the patient’s acute illness and possible contributory concurrent conditions. Non-sustained ventricular tachycardia worsened in a hemodynamically unstable, hypotensive, and tachycardic patient post-BAT product infusion. The patient’s condition deteriorated, and the patient died after 20 days. The reporting physician stated that the outcome would have been the same without BAT administration. Acute kidney injury (AKI) occurred in a patient who was admitted with botulism and septic shock and increasing creatinine 6 h prior to the BAT product infusion. The AKI onset may have been related more to sepsis and the acute illness rather than BAT product. The causality assessment of these three cases was based only on the temporal relationship between BAT product infusion and SAE onset without considering other factors. The BAT product-related SAE of distributive shock occurred in a patient with anaphylactic shock and hemodynamic instability.

We recognize that the data obtained from the registry and post-marketing safety surveillance reports have limitations due to a lack of reporting specificity to determine the diagnosis of a condition (i.e., hypersensitivity, febrile reactions, etc.). Data collection depended on healthcare professionals’ willingness to complete the forms, they were not trained in the registry protocol, and not all of them were experienced in providing AE causality assessments. This resulted in missing information. Adverse events of special interest (i.e., hypersensitivity reactions, febrile reactions, bradycardia, serum sickness, and hemodynamic instability) were specifically solicited.

In our analysis, we conservatively calculated these adverse event incidence rates using the total number of subjects treated with BAT product with a record (512). However, it may be appropriate to calculate the incidence rate using the estimated total exposure to BAT product using pre-licensure data as well as using BAT product post-licensure deployment information (1128) refer to Figure 2. Considering the two denominators, we report the BAT product-related incidence rates as a range where 78 or 6.91–15.2% of BAT product-exposed individuals had at least one BAT product-related event. Hypersensitivity, serum sickness, and anaphylaxis were determined to occur in 2.3–5.1%, 0.09–0.2%, and 0.62–1.37%, respectively, of BAT product-exposed individuals. We identified pyrexia, tachycardia, bradycardia, and blood pressure increase in 1.8–3.9%, 1.0–2.2%, 0.89–2.0%, and 0.62–1.4% of individuals, respectively, in greater than 1% of the 512–1128 individuals. Nausea, headache, and urticaria were each reported in five (0.44–0.98%) individuals. Hemodynamic instability was the only adverse event of special interest that was not reported in greater than 1% of BAT product-exposed individuals reported in 0.18–0.39%.

BAT product is indicated for the treatment of symptomatic botulism following documented or suspected exposure to botulinum neurotoxin serotypes A, B, C, D, E, F, or G in adults and pediatric patients. Each vial of BAT contains a minimum potency for serotypes A, B, C, D, E, F, and G antitoxin. The authors acknowledge there is limited product data available against novel botulinum toxin serotypes, such as BoNT/H (also called BoNT/FA or BoNT/HA). However, based on the knowledge that BoNT/H, identified as a hybrid between serotypes A and F and the recommended dose containing both antitoxins to serotypes A and F and is anticipated to contain sufficient potency to neutralize BoNT/H, but this remains to be determined.

## 4. Conclusions

The results of our analysis demonstrate that although the risk of hypersensitivity reactions including serum sickness and anaphylaxis exists with the use of BAT product, the incidence of these reactions, hypersensitivity, serum sickness, and anaphylaxis occurred in 2.3–5.1%, 0.09–0.2%, and 0.62–1.37%, respectively, of BAT product-exposed individuals, which is similar or lower than rates reported in the literature. Adverse events of special interest, i.e., pyrexia, bradycardia, in 1.8–3.9%, 0.89–2.0% were found in greater than 1% of the 512–1128 individuals. Hemodynamic instability was reported in 0.18–0.39% of BAT product-exposed individuals. The reports of myocardial infarction, ventricular tachycardia, and acute kidney injury were unexpected, and they were considered related by the reporter due to the temporal relationship and do not change the safety profile or the overall benefit-risk of the product when used according to the approved prescribing information.

These data demonstrate that for patients properly managed in an intensive care setting, the advantages of BAT product appear to outweigh potential risks in adult and pediatric patients due to morbidity and mortality of botulism.

## 5. Materials and Methods

### 5.1. Data Sources

This review follows the methods outlined in the Preferred Reporting Items for Systematic Reviews and Meta-Analyses (PRISMA) [47] and was registered with the International Prospective Register of Systematic Reviews.

Data sources included two EBCI-sponsored studies in healthy adult volunteers (BT-001 [23] and BT-002 [22]), a CDC-sponsored EAP under IND [24], a post-licensure observational patient registry (BT-010) [25], and post-marketing spontaneous and literature individual case safety reports received from licensure up to a cutoff date of 21 March 2020 (Table 1).

### 5.2. Data Eligibility Criteria

Only confirmed BAT product exposures in the pre- and post-U.S. licensure settings (i.e., BT-001, BT-002, CDC’s EAP, BT-010, U.S. CDC BAT product deployment information, ROW BAT product distribution) up to 21 March 2020 were included. Only BAT product-related AEs from the specified data sources are included in this review. Only literature articles reporting BAT product-related AEs published in the English language were included.

### 5.3. Literature Search Strategy

A PubMed^®^ search was performed to identify BAT product-related AEs reported from literature using keywords such as botulism, *Clostridium botulinum*, immune globulin, heptavalent botulism antitoxin, BAT, and antitoxin. The search strategy used is detailed in Supplementary Material Text S1.

### 5.4. Article Screening

Literature search results were evaluated independently by two EBCI reviewers to determine whether any BAT product-related AEs occurred. Any titles or abstracts referring to botulism antitoxin or BAT product use in humans between 2005 and 21 March 2020 were considered. Only articles that reported BAT product-related AEs in humans were included in the analysis. Any discordance between reviewers during abstract/title review resulted in a full-text review of the article to identify BAT product-related AEs. BAT product-related AEs from literature are included as post-marketing safety surveillance case reports.

### 5.5. Data Synthesis and Analysis

Each data source (refer to Table 1) had its own database, which contained adverse event information collected in accordance with 21CFR312.32 or 21CRF314.80/21CFR600.80 as applicable. International Council for Harmonization of Technical Requirements for Pharmaceuticals for Human Use, Clinical Safety Data Management: Definitions and Standards for Expedited Reporting (ICH E2A) [48] or World Health Organization Uppsala Monitoring Center (WHO-UMC) [49] causality assessment definitions were used by investigators, healthcare professionals, and the EBCI Pharmacovigilance (PV) physician to assess AE/SAE causality.

All EBCI-sponsored clinical study/registry AEs/SAEs were assessed for causality by investigators and healthcare professionals and were entered into a clinical database. All AEs/SAEs from post-marketing data sources were entered into Argus. Only SAEs from clinical studies are entered into Argus. The EBCI PV physician assesses causality for all AEs/SAEs that are entered into Argus. Causality assessments of “certain”, “probable”, and “possible” were considered related to BAT product. Assessments of “unlikely”, “conditional/unclassifiable”, or “unassessable/unclassifiable” were considered not related.

To perform this safety analysis, demographic and AE/SAE information from each database (Table 1) was combined into a single composite master data set. Since SAEs collected within the EBCI study and registry databases and the CDC EAP [24] were also collected in Argus, duplicate SAEs were identified and removed from the single composite master data set by using matching subject and patient identification numbers (IDs). During the duplication removal process, the MedDRA AE coding from Argus was used, and the causality assessments made by the PV physician were maintained.

### 5.6. Identification of Events of Interest

**Figure 3:**
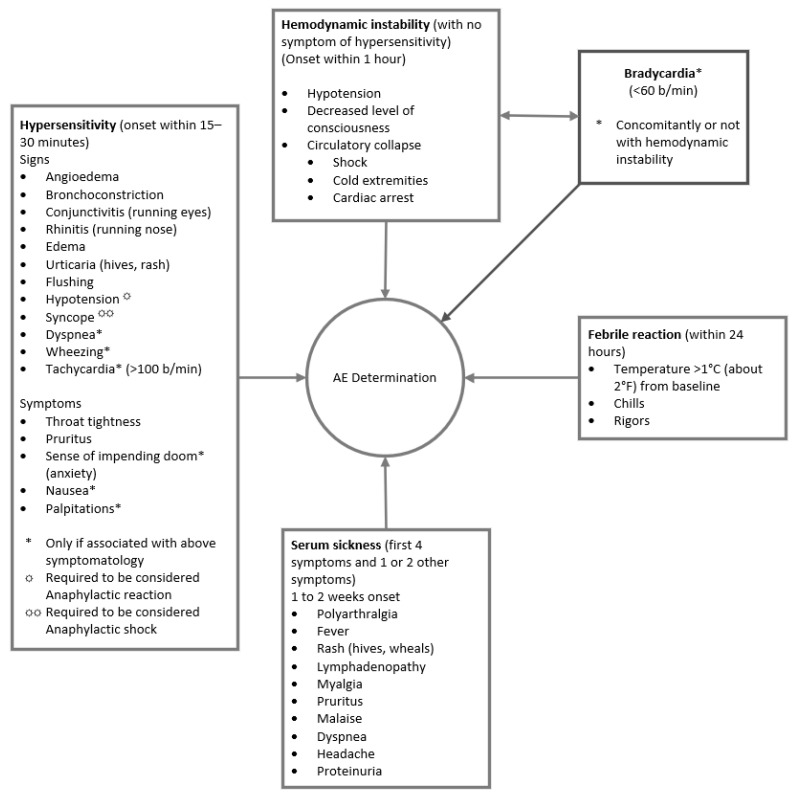
Adverse reaction algorithm was used for the evaluation of adverse events of special interest. Signs and/or symptoms of each AESI (bradycardia, hemodynamic instability, hypersensitivity, serum sickness, and febrile reactions) were defined in the algorithm. Cases of hypersensitivity were further classified as anaphylaxis based on its severity and progression.

The composite master data set was assessed for related AESI (i.e., hemodynamic instability, hypersensitivity reactions, serum sickness, febrile reactions, and bradycardia) using an adverse reaction algorithm that was developed for use in the registry (Figure 3) [25].

Since hypersensitivity reactions are not always reported as hypersensitivity in the database, we included AE terms that could be representative of hypersensitivity. According to Gell and Coombs’ classification of immunologic drug reactions, immunoglobulin E (IgE)-mediated (type I) immediate-type hypersensitivity reactions can present with signs and symptoms of redness and swelling of the skin, nasal discharge, airway narrowing, sneezing, coughing, and wheezing within minutes (mins) of allergen exposure and last up to 24–72 h [31,34]. Type III hypersensitivity reactions (i.e., immune-complex reactions) can result in serum sickness [31,34]. For cases where only hypersensitivity symptoms were reported, the PV physician used the major and minor criteria in the Brighton Collaboration case definition of anaphylaxis to determine whether the reactions reported in our master data set could be considered as anaphylaxis (Supplementary Material Text S2) [32]. Combinations of major or minor criteria or involving greater than two organ systems (one major dermatological, cardiovascular or respiratory) [32] were considered as anaphylaxis and included in the calculation of the incidence of anaphylaxis.

Febrile reactions were defined as temperature > 38.0 °C or an increase in temperature > 1 °C above baseline temperature that occurred during or within a few hours after BAT product infusion and is associated with chills [50]. Medical judgment determined the presence of febrile reaction. Hemodynamic instability was defined as a state requiring pharmacologic or mechanical support to maintain normal blood pressure or adequate cardiac output. Bradycardia was defined as a rate of <60 beats per min in adults.

### 5.7. BAT Product Exposed Population

BAT product is provided as a single dose, single-use vial. The prescribing information states that a single vial should provide sufficient drugs for a single dose in adult patients. The infant dose is 10% of the adult dose regardless of body weight; for all other pediatric patients, dosing is based on the patient weight up to 55 kg [16]. Therefore, we estimated that one BAT vial equates to one dose, and hence one patient exposure in the post-market setting.

The adverse event (AE) incidence rate for BAT product was calculated conservatively using only BAT product exposures for individuals with complete AE ascertainment (master data set—denominator B), and alternatively estimated using all BAT product exposure data from pre-licensure data as well all post-licensure deployment information (denominator A) refer to Figure 2. A subset of individuals represents those with botulism (denominator C).

### 5.8. Calculation of AE Incidence Rate

The incidence of related AEs was calculated as the number of subjects or patients reporting related AEs/SAEs (numerator) out of the total number of BAT product-exposed individuals (denominator).

## Figures and Tables

**Table 1:** Description of studies and programs used for the safety summary.

Data Source (Sponsor/Marketing Authorization Holder)	Study or Program Description	Number of BAT Product ^1^-Treated Subjects/Patients	Status
BT-001 (EBCI) [23]	Phase 1, single-center, randomized, double-blind, parallel-arm safety and PK clinical study (U.S.). A single (1 vial) or double (2 vials) dose of BAT product was administered intravenously (IV) to healthy volunteers between 19 and 52 years of age. BAT product safety assessments were based on clinical observations, AEs, and laboratory tests ^2^ following administration.	N = 20 N = 20 (double dose)	Completed
BT-002 (EBCI) [22]	Phase 1b/2a, single-center, randomized, double-blind, parallel-arm, placebo-controlled safety and pharmacodynamic clinical study (U.S.) to evaluate the safety of BAT product and its effect in preventing paralysis induced by serotype A and serotype B botulinum toxins in the extensor digitorum brevis (foot) muscle. A single dose of BAT product was administered IV to healthy volunteers between 19 and 49 years of age. BAT product safety assessments were based on clinical observations, AEs, and laboratory tests ^2^ following administration.	N = 16	Completed
EAP (CDC) [24]	This expanded-access Investigational New Drug program (“compassionate use” IND) for the investigational BAT product implemented by the U.S. CDC collected safety and clinical benefit data prospectively and through medical record reviews of patients with confirmed or suspected botulism who were treated with investigational BAT product.	N = 249 (232 adults and 17 pediatric)	Completed
Post-marketing safety surveillance (EBCI)	Monitors the safety of BAT product post-licensure. Post-marketing AEs may be received from healthcare professionals, consumers, or from literature searches. Routine pharmacovigilance activities include case processing, benefit/risk assessment, and risk management plans.	N = 755	Ongoing
BT-010 (EBCI) [25]	A post-marketing BAT product registry to evaluate the safety and clinical outcomes of pediatric and adult patients following BAT product treatment for confirmed or suspected exposure to botulinum toxin and to estimate the absolute risk of hypersensitivity/allergic reactions, including serum sickness, febrile reactions, hemodynamic instability, bradycardia, and other SAEs in patients treated with BAT product.	N = 162(153 adults and 9 pediatric)	Completed

EBCI = Emergent BioSolutions Canada Inc.; EAP = expanded-access program; U.S. = United States of America; CDC = Centers for Disease Control and Prevention; AE = adverse event; SAE = serious adverse event. ^1^ Each single-use vial of BAT product contains a minimum potency of 4500 Units (U) for serotype A antitoxin, 3300 U for serotype B antitoxin, 3000 U for serotype C antitoxin, 600 U for serotype D antitoxin, 5100 U for serotype E antitoxin, 3000 U for serotype F antitoxin, and 600 U for serotype G antitoxin. A single adult dose of BAT product is one vial, and pediatric dosing is a proportion of one vial based on body weight of the pediatric patient or 10% of adult dose regardless of body weight for infants. BAT is administered by slow intravenous (IV) infusion after dilution 1:10 in normal saline at the dose recommended in the product label. ^2^ Laboratory tests include serum chemistry, hematology, urinalysis, 12-lead ECG, viral serology, pregnancy testing (serum and urine), as well as urine drug screening collected during the study.

**Table 2:** Summary of demographics (safety population).

Statistic	Botulism (N = 294)	Healthy Volunteers (N = 56)	Other ^1^ (N = 162)	Total (N = 512)
Age Group—n (%)				
Newborn Infants (0–<28 days)	1 (0.34)	0 (0.00)	1 (0.62)	2 (0.39)
Infants/Toddlers (28–<2 years)	5 (1.70)	0 (0.00)	1 (0.62)	6 (1.17)
Children (2–<12 years)	4 (1.36)	0 (0.00)	4 (2.47)	8 (1.56)
Adolescents (12–<17 years)	5 (1.70)	0 (0.00)	5 (3.09)	10 (1.95)
Adults (18–<65 years)	225 (76.53)	56 (100.00)	96 (59.26)	377 (73.63)
Geriatric (>=65 years)	46 (15.65)	0 (0.00)	32 (19.75)	78 (15.23)
Unknown	8 (2.72)	0 (0.00)	23 (14.20)	31 (6.05)
Age ^2^ (Years)				
N	286	56	139	481
Mean (SD)	46 (18.4)	32 (9.4)	50 (20.7)	45 (19.0)
Median	45	30	53	44
Range (Min, Max)	(0, 92)	(19, 52)	(0, 88)	(0, 92)
Race—n (%)				
American Indian or Alaska Native	23 (7.82)	1 (1.79)	4 (2.47)	28 (5.47)
Asian	7 (2.38)	1 (1.79)	9 (5.56)	17 (3.32)
Black or African American	25 (8.50)	0 (0.00)	9 (5.56)	34 (6.64)
Native Hawaiian or Pacific Islander	1 (0.34)	1 (1.79)	1 (0.62)	3 (0.59)
White	131 (44.56)	49 (87.50)	72 (44.44)	252 (49.22)
Multiple Race	1 (0.34)	2 (3.57)	0 (0.00)	3 (0.59)
Not Reported	105 (35.71)	2 (3.57)	67 (41.36)	174 (33.98)
Sex—n (%)				
Female	84 (28.57)	28 (50.00)	61 (37.65)	173 (33.79)
Male	210 (71.43)	28 (50.00)	100 (61.73)	338 (66.02)
Not Reported	0 (0.00)	0 (0.00)	1 (0.62)	1 (0.20)
Ethnicity—n (%)				
Hispanic or Latino	27 (9.18)	28 (50.00)	8 (4.94)	63 (12.30)
Not Hispanic or Latino	59 (20.07)	28 (50.00)	31 (19.14)	118 (23.05)
Not Reported	208 (70.75)	0 (0.00)	123 (75.93)	331 (64.65)

N = number of subjects in the analysis population; n = number of subjects; % = n/N × 100; SD = Standard deviation. ^1^ Non-botulism final diagnosis. ^2^ Age represents subject’s age at the time of BAT product treatment.

**Table 3:** Cumulative summary tabulations of related adverse events by final diagnosis.

System Organ Class Preferred Term	Botulism N = 294		Healthy Volunteers N = 56		Other ^1^ N = 162		Total N = 512	
	Events	Subjects n (%)	Events	Subjects n (%)	Events	Subjects n (%)	Events	Subjects n (%)
OVERALL	72	46 (15.65)	32	10 (17.86)	36	22 (13.58)	140	78 (15.23)
Cardiac Disorders	13	12 (4.08)	0	0 (0.00)	11	11 (6.79)	24	23 (4.49)
Acute Myocardial Infarction	1	1 (0.34)	0	0 (0.00)	0	0 (0.00)	1	1 (0.20)
Bradycardia	5	5 (1.70)	0	0 (0.00)	5	5 (3.09)	10	10 (1.95)
Cardiac Arrest	1	1 (0.34)	0	0 (0.00)	0	0 (0.00)	1	1 (0.20)
Tachycardia	6	6 (2.04)	0	0 (0.00)	5	5 (3.09)	11	11 (2.15)
Ventricular Tachycardia	0	0 (0.00)	0	0 (0.00)	1	1 (0.62)	1	1 (0.20)
Gastrointestinal Disorders	2	2 (0.68)	6	4 (7.14)	2	2 (1.23)	10	8 (1.56)
Dysphagia	0	0 (0.00)	1	1 (1.79)	0	0 (0.00)	1	1 (0.20)
Flatulence	0	0 (0.00)	1	1 (1.79)	0	0 (0.00)	1	1 (0.20)
Lip Swelling	1	1 (0.34)	0	0 (0.00)	0	0 (0.00)	1	1 (0.20)
Nausea	0	0 (0.00)	3	3 (5.36)	2	2 (1.23)	5	5 (0.98)
Swollen Tongue	1	1 (0.34)	0	0 (0.00)	0	0 (0.00)	1	1 (0.20)
Throat Irritation	0	0 (0.00)	1	1 (1.79)	0	0 (0.00)	1	1 (0.20)
General Disorders and Administration Site Conditions	14	13 (4.42)	6	4 (7.14)	11	9 (5.56)	31	26 (5.08)
Chest Discomfort	0	0 (0.00)	1	1 (1.79)	1	1 (0.62)	2	2 (0.39)
Chills	0	0 (0.00)	0	0 (0.00)	1	1 (0.62)	1	1 (0.20)
Feeling Cold	0	0 (0.00)	1	1 (1.79)	0	0 (0.00)	1	1 (0.20)
Feeling Jittery	0	0 (0.00)	0	0 (0.00)	1	1 (0.62)	1	1 (0.20)
Edema	1	1 (0.34)	0	0 (0.00)	1	1 (0.62)	2	2 (0.39)
Pain	0	0 (0.00)	1	1 (1.79)	0	0 (0.00)	1	1 (0.20)
Pyrexia	12	12 (4.08)	2	1 (1.79)	7	7 (4.32)	21	20 (3.91)
Swelling	1	1 (0.34)	1	1 (1.79)	0	0 (0.00)	2	2 (0.39)
Immune System Disorders	8	8 (2.72)	0	0 (0.00)	3	3 (1.85)	11	11 (2.15)
Anaphylactic Reaction	1	1 (0.34)	0	0 (0.00)	2	2 (1.23)	3	3 (0.59)
Anaphylactic Shock	1	1 (0.34)	0	0 (0.00)	0	0 (0.00)	1	1 (0.20)
Hypersensitivity	3	3 (1.02)	0	0 (0.00)	1	1 (0.62)	4	4 (0.78)
Serum Sickness	1	1 (0.34)	0	0 (0.00)	0	0 (0.00)	1	1 (0.20)
Serum Sickness-Like Reaction	2	2 (0.68)	0	0 (0.00)	0	0 (0.00)	2	2 (0.39)
Injury, Poisoning and Procedural Complications	1	1 (0.34)	0	0 (0.00)	0	0 (0.00)	1	1 (0.20)
Drug Administration Error	1	1 (0.34)	0	0 (0.00)	0	0 (0.00)	1	1 (0.20)
Investigations	11	10 (3.40)	2	1 (1.79)	4	3 (1.85)	17	14 (2.73)
Blood Fibrinogen Increased	0	0 (0.00)	1	1 (1.79)	0	0 (0.00)	1	1 (0.20)
Blood Pressure Decreased	3	3 (1.02)	0	0 (0.00)	0	0 (0.00)	3	3 (0.59)
Blood Pressure Increased	4	4 (1.36)	0	0 (0.00)	3	3 (1.85)	7	7 (1.37)
Body Temperature Increased	2	2 (0.68)	1	1 (1.79)	0	0 (0.00)	3	3 (0.59)
Heart Rate Decreased	1	1 (0.34)	0	0 (0.00)	0	0 (0.00)	1	1 (0.20)
Heart Rate Increased	0	0 (0.00)	0	0 (0.00)	1	1 (0.62)	1	1 (0.20)
White Blood Cell Count Increased	1	1 (0.34)	0	0 (0.00)	0	0 (0.00)	1	1 (0.20)
Musculoskeletal and Connective Tissue Disorders	2	1 (0.34)	2	1 (1.79)	0	0 (0.00)	4	2 (0.39)
Arthralgia	1	1 (0.34)	0	0 (0.00)	0	0 (0.00)	1	1 (0.20)
Musculoskeletal Stiffness	0	0 (0.00)	2	1 (1.79)	0	0 (0.00)	2	1 (0.20)
Myalgia	1	1 (0.34)	0	0 (0.00)	0	0 (0.00)	1	1 (0.20)
Nervous System Disorders	1	1 (0.34)	7	5 (8.93)	0	0 (0.00)	8	6 (1.17)
Headache	0	0 (0.00)	7	5 (8.93)	0	0 (0.00)	7	5 (0.98)
Seizure	1	1 (0.34)	0	0 (0.00)	0	0 (0.00)	1	1 (0.20)
Psychiatric Disorders	1	1 (0.34)	0	0 (0.00)	1	1 (0.62)	2	2 (0.39)
Agitation	0	0 (0.00)	0	0 (0.00)	1	1 (0.62)	1	1 (0.20)
Anxiety	1	1 (0.34)	0	0 (0.00)	0	0 (0.00)	1	1 (0.20)
Renal and Urinary Disorders	2	2 (0.68)	0	0 (0.00)	1	1 (0.62)	3	3 (0.59)
Acute Kidney Injury	1	1 (0.34)	0	0 (0.00)	0	0 (0.00)	1	1 (0.20)
Hematuria	1	1 (0.34)	0	0 (0.00)	0	0 (0.00)	1	1 (0.20)
Urinary Retention	0	0 (0.00)	0	0 (0.00)	1	1 (0.62)	1	1 (0.20)
Respiratory, Thoracic and Mediastinal Disorders	0	0 (0.00)	1	1 (1.79)	1	1 (0.62)	2	2 (0.39)
Bronchospasm	0	0 (0.00)	0	0 (0.00)	1	1 (0.62)	1	1 (0.20)
Pharyngolaryngeal Pain	0	0 (0.00)	1	1 (1.79)	0	0 (0.00)	1	1 (0.20)
Skin and Subcutaneous Tissue Disorders	10	10 (3.40)	8	4 (7.14)	2	2 (1.23)	20	16 (3.13)
Dermatitis Allergic	1	1 (0.34)	0	0 (0.00)	0	0 (0.00)	1	1 (0.20)
Erythema	1	1 (0.34)	0	0 (0.00)	0	0 (0.00)	1	1 (0.20)
Hyperhidrosis	2	2 (0.68)	1	1 (1.79)	0	0 (0.00)	3	3 (0.59)
Night Sweats	1	1 (0.34)	0	0 (0.00)	0	0 (0.00)	1	1 (0.20)
Pruritus	0	0 (0.00)	1	1 (1.79)	0	0 (0.00)	1	1 (0.20)
Pruritus Generalized	0	0 (0.00)	1	1 (1.79)	0	0 (0.00)	1	1 (0.20)
Rash	3	3 (1.02)	0	0 (0.00)	1	1 (0.62)	4	4 (0.78)
Rash Erythematous	0	0 (0.00)	0	0 (0.00)	1	1 (0.62)	1	1 (0.20)
Skin Disorder	0	0 (0.00)	1	1 (1.79)	0	0 (0.00)	1	1 (0.20)
Urticaria	2	2 (0.68)	4	3 (5.36)	0	0 (0.00)	6	5 (0.98)
Vascular Disorders	7	5 (1.70)	0	0 (0.00)	0	0 (0.00)	7	5 (0.98)
Distributive Shock	1	1 (0.34)	0	0 (0.00)	0	0 (0.00)	1	1 (0.20)
Flushing	1	1 (0.34)	0	0 (0.00)	0	0 (0.00)	1	1 (0.20)
Hemodynamic Instability	2	2 (0.68)	0	0 (0.00)	0	0 (0.00)	2	2 (0.39)
Hypotension	3	3 (1.02)	0	0 (0.00)	0	0 (0.00)	3	3 (0.59)

N = number of subjects in the analysis population; n = number of subjects; % = n/N × 100. Subjects with more than one event in a category are counted once in each category. ^1^ Non-botulism final diagnosis.

**Table 4:** Cumulative summary tabulations of related serious adverse events by final diagnosis.

	Botulism N = 294		Healthy Volunteers N = 56		Other ^1^ N = 162		Total N = 512	
System Organ Class Preferred Term	Events	Subjects n (%)	Events	Subjects n (%)	Events	Subjects n (%)	Events	Subjects n (%)
OVERALL	9	7 (2.38)	0	0 (0.00)	4	4 (2.47)	13	11 (2.15)
Cardiac Disorders	1	1 (0.34)	0	0 (0.00)	1	1 (0.62)	2	2 (0.39)
Acute Myocardial Infarction	1	1 (0.34)	0	0 (0.00)	0	0 (0.00)	1	1 (0.20)
Ventricular Tachycardia	0	0 (0.00)	0	0 (0.00)	1	1 (0.62)	1	1 (0.20)
Immune System Disorders	4	4 (1.36)	0	0 (0.00)	3	3 (1.85)	7	7 (1.37)
Anaphylactic Reaction	1	1 (0.34)	0	0 (0.00)	2	2 (1.23)	3	3 (0.59)
Anaphylactic Shock	1	1 (0.34)	0	0 (0.00)	0	0 (0.00)	1	1 (0.20)
Hypersensitivity	2	2 (0.68)	0	0 (0.00)	1	1 (0.62)	3	3 (0.59)
Renal and Urinary Disorders	1	1 (0.34)	0	0 (0.00)	0	0 (0.00)	1	1 (0.20)
Acute Kidney Injury	1	1 (0.34)	0	0 (0.00)	0	0 (0.00)	1	1 (0.20)
Vascular Disorders	3	2 (0.68)	0	0 (0.00)	0	0 (0.00)	3	2 (0.39)
Distributive Shock	1	1 (0.34)	0	0 (0.00)	0	0 (0.00)	1	1 (0.20)
Hemodynamic Instability	2	2 (0.68)	0	0 (0.00)	0	0 (0.00)	2	2 (0.39)

N = number of subjects in the analysis population; n = number of subjects; % = n/N × 100; Subjects with more than one event in a category are counted once in each category. ^1^ Non-botulism final diagnosis.

## Data Availability

The data presented in this review are available in the supplementary material.

## Supplementary Materials

The following supporting information can be downloaded at: https://www.mdpi.com/article/10.3390/toxins14010019/s1, Text S1. Search terms and strategies (DOCX); Text S2. Brighton Collaboration case definition of anaphylaxis (DOCX2); Text S3. Assessment of BAT product-related hypersensitivity reactions (DOCX3).
