# Supplementary material for: Use of Botulism Antitoxin Heptavalent (A, B, C, D, E, F, G)—(Equine) (BAT®) in Clinical Study Subjects and Patients: A 15-Year Systematic Safety Review
Source: Toxins (Basel). 2021 Dec 27;14(1):19. doi: 10.3390/toxins14010019 (PMC8778610; doi:10.3390/toxins14010019)
Supplement: Supplementary file 1 [file toxins-14-00019-s001.zip › toxins-1494991-supplementary.pdf]

toxins MDPI

Supplementary Materials:Use of Botulism Antitoxin Heptavalent (A, B, C, D, E, F, G)—(Equine) (BAT®) in Clinical Study Subjects and Patients: A 15-Year Systematic Safety Review

Geraldine S. Parrera, Hugo Astacio, Priya Tunga, Deborah M. Anderson, Christine L. Hall and Jason S. Richardson

Supplementary Text S1

Search terms and strategies

MEDLINE®/PubMed®

("botulism"[MeSH Terms] OR "botulism"[All Fields] OR "clostridium botulinum"[All Fields]) AND ((("immune"[All Fields]) AND ("globulins"[MeSH Terms] OR "globulins"[All Fields] OR "globulin"[All Fields] OR "globulin fragments"[All Fields])) OR "antitoxin"[All Fields]) Filters: from 2005/1/1 - 2020/3/22

MeSH = Medical Subject Headings (MeSH®)

Supplementary Text S2

Brighton Collaboration

Table 5. Brighton Collaboration Case Definition of Anaphylaxis.

Level of Diagnostic Certainty	Case Definition
For all levels	Anaphylaxis is a clinical syndrome characterized by:<br>sudden onset AND<br>rapid progression of signs and symptoms AND<br>involving multiple (≥2) organ systems, as follows
Level 1	≥1 major dermatological AND<br>≥1 major cardiovascular AND/OR ≥1 major respiratory criterion
Level 2	≥1 major cardiovascular AND ≥1 major respiratory criterion OR<br>≥1 major cardiovascular OR respiratory criterion AND<br>≥1 minor criterion involving ≥1 different system (*other than* cardiovascular or respiratory systems) OR<br>(≥1 major dermatologic) AND (≥1 minor cardiovascular AND/OR minor respiratory criterion)
Level 3	≥1 minor cardiovascular OR respiratory criterion AND<br>≥1 minor criterion from each of ≥2 different systems/categories

The case definition should be applied when there is no clear alternative diagnosis for the reported event to account for the combination of symptoms.

Extracted from Ruggeberg *et al*. [32].

Table 6 Major and Minor Criteria Used in the Brighton Collaboration Case Definition of Anaphylaxis

Organ system	Minor Criteria	Major Criteria
Dermatologic or mucosal	• generalized pruritus without skin rash<br>• generalized prickle sensation<br>• localized injection site urticaria<br>• red and itchy eyes	• generalized urticaria (hives) or generalized erythema<br>• angioedema (not hereditary), localized or generalized<br>• generalized pruritus with skin rash
Cardiovascular	• Reduced peripheral circulation as indicated by the combination of at<br>• least 2 of the following:<br>o tachycardia<br>o a capillary refill time of >3 s without hypotension<br>o a decreased level of consciousness	• measured hypotension<br>• clinical diagnosis of uncompensated shock, indicated by the combination of at least 3 of the following:<br>o tachycardia<br>o capillary refill time >3 s<br>o reduced central pulse volume<br>o decreased level of consciousness or loss of consciousness
Respiratory	• persistent dry cough<br>• hoarse voice<br>• difficulty breathing without wheeze or stridor<br>• sensation of throat closure<br>• sneezing, rhinorrhea	• bilateral wheeze (bronchospasm)<br>• stridor<br>• upper airway swelling (lip, tongue, throat, uvula, or larynx)<br>• respiratory distress–2 or more of the following:<br>o tachypnoea

**Organ system**	**Minor Criteria**	**Major Criteria**
		○ increased use of accessory respiratory muscles (sternocleidomastoid, intercostals, etc.)<br>○ recession<br>○ cyanosis<br>○ grunting
Gastrointestinal	• diarrhoea<br>• abdominal pain<br>• nausea<br>• vomiting	Not applicable
Laboratory	• Mast cell tryptase elevation > upper normal limit	Not applicable

Adapted from Ruggeberg *et al*. [32].

Reference

32. Ruggeberg, J.U.; Gold, M.S.; Bayas, J.M.; Blum, M.D.; Bonhoeffer, J.; Friedlander, S.; de Souza Brito, G.; Heininger, U.; Imoukhuede, B.; Khamesipour, A., et al. Anaphylaxis: case definition and guidelines for data collection, analysis, and presentation of immunization safety data. *Vaccine* **2007**, *25*, 5675-5684, doi:10.1016/j.vaccine.2007.02.064.

Supplementary Text S3

Assessment of BAT Product Related Hypersensitivity Reactions

Case Source[1]	Subset	Onset	Adverse Event (MedDRA PT)	Description	Anaphylaxis Determination (by PV Physician)
Clinical Study[2] Subject 1	Healthy Volunteer	During infusion	Urticaria, Pruritus, Skin disorder	Infusion stopped 52 mins after onset of BAT product infusion. Treated with Epinephrine and Diphenhydramine.	Case presented two minor criteria (urticaria was not generalized) of the same dermatological class. Not anaphylaxis. Probable anaphylaxis was prevented.
Clinical Study[2] Subject 2	Healthy Volunteer	Same day of infusion	Pruritus generalised, Urticaria	Subject developed itchy welts 1.5-4 cm in diameter on scalp and mandible.	Case presented one major (pruritus generalized) and one minor criterion (urticaria was not generalized) of the dermatological class. Not anaphylaxis.
Clinical Study[2] Subject 3	Healthy Volunteer	Same day of infusion	Swelling	Temporal swelling in the right arm 42 mins after infusion onset, Resolved after one hour.	Assumed angioedema, a major criterion of the dermatological class. Not anaphylaxis.
Clinical Study[2] Subject 4	Healthy Volunteer	Same day of infusion	Throat irritation	No other information received; conservatively considered associated to hypersensitivity.	Assumed angioedema, a major criterion of dermatological. Not anaphylaxis.
Clinical Study[3] Subject 5	Healthy Volunteer	During infusion	Urticaria	Infusion was immediately terminated and treated with Benadryl and Solumedrol.	Urticaria was not generalized, considered as one minor of the dermatological criterion. Possible medication prevented progression to anaphylaxis. Not anaphylaxis.
Clinical Study[4] Subject 6	Not botulism	During infusion	Bronchospasm, Tachycardia	Subject treated with Solu-Medrol and Benadryl. BAT product infusion rate decreased. Subject received full dose.	Case presented one major respiratory and one minor cardiovascular criterion. Case met level 2 of the diagnostic certainty criteria of anaphylaxis.
Clinical Study[4] Subject 7	Botulism	12 days after infusion	Serum sickness	Delayed reaction. Serum sickness associated with myalgia and arthralgia.	Serum sickness is not an acute hypersensitivity. Not anaphylaxis.
Clinical Study[4] Subject 8	Botulism	Day after infusion	Rash	No other information	Case presented one minor criterion of the dermatological class. Not anaphylaxis.

**Case Source[1]**	**Subset**	**Onset**	**Adverse Event (MedDRA PT)**	**Description**	**Anaphylaxis Determination (by PV Physician)**
Clinical Study[4] Subject 9	Botulism	Day after infusion	Rash	No other information	Case presented one minor criterion of the dermatological class. Not anaphylaxis.
Clinical Study[4] Subject 10	Not botulism	Day after infusion	Rash	No other information	Case presented one minor criterion of the dermatological class. Not anaphylaxis.
Patient Registry Case 1	Botulism	During infusion	Serum sickness-like reaction	Patient developed "Serum sickness-like reaction". Infusion was interrupted halfway and was restarted after 50 mins. Infusion was completed without problems.	Hypersensitivity diagnosis reported. Not anaphylaxis.
Patient Registry Case 2	Botulism	Same day of infusion	Hypersensitivity	Mild rash and flushing after the infusion. Resolved spontaneously. Reported as diagnosis.	Hypersensitivity diagnosis reported. The rash presented is one minor dermatological criterion. Not anaphylaxis.
Patient Registry Case 3	Botulism	Same day of infusion	Dermatitis allergic	Patient developed an allergic skin reaction - generalized erythema (red warm area to left forearm, bilateral shoulders and back) within an hour after infusion onset. Infusion was stopped.	Case met one major of dermatological. Not anaphylaxis.
Patient Registry Case 4	Botulism	Same day of infusion	Serum sickness-like reaction	Reported as diagnosis.	Hypersensitivity diagnosis reported. Not anaphylaxis.
Patient Registry Case 5	Botulism	Three days after infusion	Rash	Mild rash on the back, buttocks and thighs.	Case presented one minor criterion of the dermatological criterion. Not anaphylaxis
Patient Registry Case 6	Not botulism	During infusion	Anaphylactic reaction	Anaphylactic reaction occurred within one hour after infusion started. Infusion stopped.	Anaphylaxis diagnosis reported. Further anaphylaxis assessment not required.
Spontaneous Case 7	Botulism	Same day of infusion	Anaphylactic shock, Hemodynamic instability, Hypotension, Lip swelling,	Anaphylactic shock occurred a few hours after infusion. Hemodynamic instability was due to anaphylaxis.	Anaphylaxis diagnosis reported. Further anaphylaxis assessment not required.

Case Source[1]	Subset	Onset	Adverse Event (MedDRA PT)	Description	Anaphylaxis Determination (by PV Physician)
			Swelling, Distributive shock		
Spontaneous Case 8	Botulism	Same day of infusion	Swollen tongue	Considered as angioedema.	Case presented one major criterion of the dermatological class. Not anaphylaxis.
Spontaneous Case 9	Botulism	During infusion	Hypersensitivity, Urticaria	Allergic reaction occurred within one hour after infusion onset. Infusion was re-started after 16 hours, and completed without problems.	Hypersensitivity diagnosis reported. Urticaria was not generalized, considered as one minor of the dermatological criterion. Not anaphylaxis.
Spontaneous Case 10	Botulism	During infusion	Anaphylactic reaction	A patient developed anaphylaxis during BAT-product infusion. The infusion was stopped.	Anaphylaxis diagnosis reported. Further anaphylaxis assessment not required.
Spontaneous Case 11	Botulism	Four hours after infusion	Hypersensitivity, Hypotension	Possible anaphylaxis. Reported as life threatening condition.	Case met level 2 of diagnostic certainty criteria of anaphylaxis.
Spontaneous Case 12	Not botulism	Same day of infusion	Rash Erythematous	Mild erythematous rash 15 hours after infusion, resolved spontaneously and lasted one min. Transient reaction, self resolved.	Rash is one minor criterion of the dermatological class. Not anaphylaxis.
Spontaneous Case 13	Botulism	Fifteen days after infusion	Urticaria	Delayed reaction two weeks post infusion, possibly caused by Clindamycin.	It doesn't meet the term of acute. Urticaria was not generalized, considered as minor criterion of the dermatological class. Not anaphylaxis.
Spontaneous Case 14	Botulism	Same day of infusion	Flushing	Self limited reaction several hours after infusion; healthcare provider was unsure if it was an allergic reaction.	Not anaphylaxis
Spontaneous Case 15	Not botulism	During the infusion	Anaphylactic reaction	Hypersensitivity reaction with itching, flushing, brief hypotension. The infusion was stopped, treated with diphenhydramine, Solumedrol and normal	Anaphylaxis diagnosis reported. Further anaphylaxis assessment not required.

**Case Source**[1]	**Subset**	**Onset**	**Adverse Event (MedDRA PT)**	**Description**	**Anaphylaxis Determination (by PV Physician)**
				saline. BAT product infusion was completed. The symptoms did not appear during the re-challenge of the product.	
Spontaneous Case 16	Not botulism	During the infusion	Hypersensitivity	The patient developed tingling in her vaginal area with inflammation around the peri-vulvar area, rash and hypotension during the infusion. The infusion was stopped. She was treated with normal saline and Benadryl. BAT product infusion was resumed at a rate of 0.5 mL/min for about 30 mins, then increased the rate to 1 mL/min, 40 mins later, the infusion was stopped with 10 cc BAT left to infuse as the patient again developed hypotension and diffuse hives. She was given another bolus of normal saline and IV Benadryl as well as Decadron 4 mg IV.	Hypersensitivity diagnosis reported. The associated hypersensitivity symptoms meet one major of cardiovascular and one major and one minor of dermatological criterion or level 1 of the diagnostic certainty criteria of anaphylaxis.

[1] One case refers to one individual.
[2] From EBCI sponsored clinical study BT-001.
[3] From EBCI sponsored clinical study BT-002.
[4] From CDC's EAP.
